# Editorial: Insights into the effectiveness of exercise/lifestyle recommendations in primary care

**DOI:** 10.3389/fmed.2023.1286796

**Published:** 2023-10-04

**Authors:** Patricia C. Heyn

**Affiliations:** Center for Optimal Aging, Marymount University, Arlington, VA, United States

**Keywords:** exercise prescription, primary care, lifestyle medicine, physical activity recommendations, clinical exercise, health promotion, diverse patients

Exercise and lifestyle modifications have established themselves as the pillars of primary care, proving to be potent weapons in the war against non-communicable diseases. With the World Health Organization (WHO) naming physical inactivity as a leading risk factor for global mortality (1), it is clear and critical that we need to move to efficacious, sustainable, and feasible incorporation of exercise prescriptions into primary care practice (2).

There is extensive research highlighting the wide-ranging benefits of physical activity, spanning from mitigating the risks of cardiovascular diseases, diabetes, and stroke, as well as mental health conditions (3) including Alzheimer's disease (4). Furthermore, it has been shown to enhance the quality of life during cancer treatments (5). Despite this wealth of evidence supporting the significant health benefits associated with exercise, it remains concerning that approximately 27.5 % of adults globally (6) and 81% of adolescents (7) do not meet the recommendations for aerobic exercise, as outlined in the 2010 Global Recommendations on Physical Activity for Health report (8). This issue could possibly be attributed to the absence of consensus and consistent scientific interpretations concerning the factors and mechanisms that give rise to variations in individual responses to exercise training (9), specifically when we consider the variations in clinical conditions, symptoms, and diseases (9–11) and aging (12). This lack of clarity extends to the factors moderating exercise response (9), all of which are pivotal in striving to optimize the clinical impact of exercise prescription (11). Acquiring a deeper understanding of these factors is vital as it would facilitate the clinical adoption of precise and individualized exercise prescriptions (13).

Considering this, primary care professionals across the world are progressively incorporating exercise-based lifestyle modifications, (14) particularly physical activity, into their daily routines (15). The trend involves prescribing gym memberships, using technology and apps, and initiatives centered around promoting exercise's positive influence on population health (16). Nevertheless, the successful implementation, adoption, and completion of tailored exercise prescription plans face several impediments (9, 14). Thus, the primary focus of the investigation should center on evaluating the effectiveness of exercise and lifestyle prescriptions in enhancing patient outcomes within the realm of primary care (2, 10). While synthesis methodology reviews (4, 5, 15) have pointed to a positive impact on patient health outcomes, there is a distinct need for more comprehensive and intricate studies to elucidate the specifics of this impact (16). Further research efforts should be directed toward pinpointing the elements that characterize successful interventions (10) and discerning which factors contribute most significantly to fostering positive outcomes (5, 16).

Addressing the evaluation of physical activity within the context of primary care practice presents a noteworthy challenge (2). The implementation of standardized, culturally sensitive, and objective tools for assessing levels of physical activity is essential (1, 17). Such tools ensure a precise representation of patients' lifestyle habits, thus paving the way for the development of more personalized and efficacious exercise prescriptions (17). The complexities within this realm of scientific exploration are further heightened when considering the management of neurological diseases through exercise prescriptions (4, 10, 14). Existing research demonstrates promising outcomes, particularly in conditions such as Alzheimer's (4), Parkinson's disease, and multiple sclerosis (18). However, there is a clear need for an in-depth exploration of various prescription modalities to both maximize benefits and mitigate potential risks associated with managing neurological diseases through exercise interventions (14).

In addition, adherence rates continue to fall below satisfactory levels, underscoring the imperative need for the development of strategies aimed at bolstering acceptance and participation (15). Central to this endeavor is the need to effectively understand the hurdles faced by patients, especially from underrepresented backgrounds (19), in adhering to exercise plans, coupled with the creation of systems that cultivate motivation, sustained engagement, and perseverance (20). Establishing effective communication between patients and physicians stands as a pivotal factor in the implementation of an exercise plan (17). The refinement of this interaction requires concentrated efforts, encompassing clear, consistent messaging and an approach that places the patient at the center, as this approach has demonstrated the most notable success (2). Moreover, it is crucial to acknowledge the presence of confounding factors that can influence patients' adherence to exercise plans (9). Crafting inclusive and adaptable strategies is imperative to accommodate these variables effectively (19).

Furthermore, illustrating instances of successful integration of exercise recommendations within real-world settings can serve as practical models for future endeavors (16). Collaborations between clinical and community settings, involving allied health professionals such as exercise physiologists, yield valuable insights and facilitate the multidisciplinary approach necessary for the advancement of patient outcomes (17, 21). While the integration of exercise and lifestyle recommendations into primary care presents its share of challenges (13, 15), it possesses the potential to revolutionize the landscape of primary health care (11). The journey to optimize this facet of healthcare delivery is already underway (2, 8, 10), and with sustained research efforts, collaborative initiatives, and unwavering dedication, there exists a profound opportunity to enact a substantial positive impact on global health (1). The advantages of exercise and lifestyle recommendations within primary care are extensive, spanning a range of diseases and conditions (2, 16, 17). This editorial highlights 11 recent research papers that contribute to the expanding landscape of this crucial subject (Zhang et al.; Hu et al.; Liu Y. et al.; Cheng et al.; Mainous et al.; Lin et al.; Wattanapisit et al.; Liu Z. et al.; Feng et al.; Felemovicius et al.; Heyn et al.).

Zhang et al. in their paper “*Exercise for Neuropathic Pain: A Systematic Review and Expert Consensus*” emphasize that a proper exercise program can serve as an effective alternative treatment or complementary therapy for most patients with neuropathic pain (NP). This consensus provides actionable recommendations for clinicians and policymakers alike in formulating exercise prescriptions to treat NP.

A population-based study by Hu et al. evaluated all-cause mortality and cardiovascular mortality in the Guangzhou Heart Study (GZHS), an ongoing prospective population-based cohort study in South China. Specifically, they investigated the effect of the Healthy Lifestyle Index and lifestyle patterns on the risk of mortality in a large Chinese population. Their study results suggest that accumulative dimensions of a healthy lifestyle can lower the risk of death, and adherence to the healthy lifestyle pattern was associated with non-smoking and low-level alcohol consumption which reduces the risk of all-cause mortality. These findings highlight the need to consider multi-dimensional lifestyle approaches when developing health promotion strategies.

The clinical trial “*Efficacy of electro-acupuncture in postpartum women with diastasis recti abdominis: A randomized controlled clinical trial*” by Liu Y. et al. evaluated the long-term efficacy and safety of electro-acupuncture (EA) in treating diastasis rectus abdominis (DRA) during postpartum. The study results showed that AE treatment improved multiple health parameters and symptoms related to Diastasis Recti Abdominis (DRA), displaying lasting effects up to 26 weeks postpartum.

Furthermore, Cheng et al. investigated the effectiveness and response of a multidisciplinary Workplace health promotion (WHP). They used a retrospective cohort sample of healthcare workers participating in a multidisciplinary WHP program in five healthcare facilities. The 20-week intervention included exercise classes, nutrition consultation, and behavioral education followed by anthropometrics, body composition, and physical fitness (PF) measures. Their study results demonstrated that a multidisciplinary WHP could enhance anthropometric and physical fitness profiles among healthcare workers.

Mainous et al. evaluated the relationship between depression-high depressive symptomatology and adherence to lifestyle interventions among patients with prediabetes. They analyzed the 2017-20 20 National Health and Nutrition Examination Survey (NHANES), a nationally representative population of U.S. adults. Their findings showed that depression-high depressive symptomatology decreases the likelihood of adherence to exercise-based lifestyle recommendations among patients with a confirmed diagnosis of prediabetes underscoring the interplay between mental health and lifestyle changes.

A randomized trial by Lin et al. conducted a randomized controlled trial with 66 full-term infants with eczema randomly assigned to an eczema control (EC) group and an eczema with MPIM (EM) group as compared to a healthy control (HC) group. The mothers in the EC group received the instruction of routine care, while the mothers in the EM group applied massage on the infants plus receiving the same instruction of the routine care. HC group received no specific intervention. Compared with the EC group, the EM group showed significantly lower scores on eczema outcomes supporting the reduction of infantile eczema, along with relieving maternal anxiety and depression.

An intriguing opinion paper by Wattanapisit et al. highlights the importance of training, integrating, and applying knowledge and skills pertaining to PA promotion in clinical settings with the goal of covering several aspects of sport and exercise science. They remind us that PCPs are usually not specialists in these subject areas, and it is essential to supplement and provide knowledge for proper clinical practices. They include the collective perspective of experts in the field.

Liu Z. et al. conducted a systematic review and meta-analysis to evaluate the effects of a traditional Chinese Qigong therapy called Baduanjin which is characterized by symmetrical body posture and actions, breathing control, meditative state, and concentration. They reviewed randomized controlled trials evaluating Baduanjin therapy's effects on neck pain and functional movement in older individuals. Although the results of the synthesis methods support Baduanjin therapy as a safe and positive treatment for neck pain in older individuals, they called for caution as more studies are needed to validate and support its benefit.

Feng et al. through a population-based study in Taiwan, investigated whether physical activity (PA) is associated with a reduced risk of hemorrhagic stroke (HS). Their study supports the beneficial effect of PA on reducing HS risk. However, high-PA did not appear to have a greater protective effect than low-PA in diabetes and hypertension outcomes. Thus, their conclusions support that even <90 min of PA per week might be beneficial in reducing HS risk. They also note that recommendations based on low PA levels are more likely achievable and sustainable across the general population. Additionally, personalized recommendations, based on pre-existing comorbidities, may help optimize the beneficial effects of PA on HS prevention.

The SOOTHER Trail by Felemovicius et al. evaluated a novel composite topical Lidocaine agent treatment for Pruritus ani, or rectal or anal itch, which is a common perianal disorder that affects approximately five percent of the population of the developed world. The SOOTHER Trial showed efficacy in providing rapid and effective relief of pruritus ani in an ambulatory population.

Finally, a study by a research team (Heyn et al.) from the University of Colorado School of Medicine and Colorado Children's Hospital underscores the importance of lifelong monitoring of children with disabilities to identify and modify disease-induced risk factors through lifestyle interventions. Their study, “*The association between isometric strength and cognitive function in adults with cerebral palsy*,” supports using simple tests like hand grip strength to evaluate early signs of frailty and neurocognitive decline in adults with Cerebral Palsy.

In summary, when considering the results of the studies from this Research Topic collectively (Zhang et al.; Hu et al.; Liu Y. et al.; Cheng et al.; Mainous et al.; Lin et al.; Wattanapisit et al.; Liu Z. et al.; Feng et al.; Felemovicius et al.; Heyn et al.), it becomes evident that they underscore the pivotal significance of exercise and lifestyle modifications within primary care. Through their findings, they shed light on the multifaceted roles of these interventions across various health contexts. As we continue to navigate the intricacies involved in implementing efficacious exercise prescriptions and clinical physical activity recommendations in primary care settings, these studies serve as valuable resources that enrich our comprehension and improve our ability to promote healthier lifestyles effectively for diverse patients within the realm of primary care.

## Author contributions

PH: Writing—original draft, Writing—review and editing.
